# Paper-Cut Flexible Multifunctional Electronics Using MoS_2_ Nanosheet

**DOI:** 10.3390/nano9070922

**Published:** 2019-06-26

**Authors:** Dong Yang, Hao Wang, Shenglin Luo, Changning Wang, Sheng Zhang, Shiqi Guo

**Affiliations:** 1 School of Chemistry and Materials Science, University of Science and Technology of China, Hefei 230026, China; 2 Athioula A. Martinos Center for Biomedical Imaging, Department of Radiology, Massachusetts General Hospital, Harvard Medical School, Charlestown, MA 02129, USA; 3 Micro/Nano Technology Center, Tokai University, 4-1-1 Kitakaname, Hiratsuka-city, Kanagawa 259-1292, Japan; 4 School of Engineering and Applied Science, The George Washington University, Washington, DC 20052, USA; 5 John A. Paulson School of Engineering and Applied Sciences, Harvard University, Cambridge, MA 02138, USA

**Keywords:** highly stretchable electronics, Chinese traditional culture, paper-cut, bioelectronics, 2D material, flexible electronics

## Abstract

Art and science represent human creativity and rational thinking, respectively. When the two seemingly opposite fields are intertwined, there is always a life-changing spark. In particular, the integration of ancient traditional Chinese art into the latest electronic devices is always been an unexcavated topic. Fabricating two-dimensional material with a tensile strain less than 3% with an ultimate global stretch has been an important problem that plagues the current flexible electronics field. The current research is limited to material in small scale, and it is always necessary to develop and extend large-sized flexible electronic systems. Here, inspired by the traditional Chinese paper-cut structure, we present a highly deformable multifunctional electronic system based on the MoS_2_ nanosheet. In this work, we first demonstrate how the traditional paper-cut structure can open the view of flexible electronics. In order to obtain a large area of MoS_2_ with excellent performance, we use a metal-assisted exfoliation method to transfer MoS_2_, followed by fabricating a field effect transistor to characterize its excellent electrical properties. Two photodetectors and a temperature sensor are produced with good performance. The mechanical simulation proves that the structure has more advantages in stretchability than other typical paper-cut structures. From the experimental and mechanical point of view, it is proved that the device can work stably under high deformation. We finally show that the device has broad application prospects in highly deformed organs, tissues, and joints. These findings set a good example of traditional Chinese culture to guide innovation in the field of electronic devices.

## 1. Introduction

The rapid development of precision healthcare has set off a revolutionary wave in individualized diagnosis and treatment. One of the most important aspects is the tracking and collection of various information from the human body. For example, various wearable electronic devices such as watches, wristbands, and shoes have been invented for real-time signal collection [1,2,3,4]. However, due to the limitation of the fabrication process, they cannot be seamlessly laminated on human skin and organs with complex surfaces, which will affect the accuracy of the information collected. The development of ultra-thin flexible electronic devices opens up the possibility of monitoring biological information on physiological tissues with complex curved surfaces and structures. It can detect signals including temperature, optical, PH, blood sugar on the cerebral cortex, eyeballs, skin, and other tissues and organs [5,6,7,8,9,10,11]. Physiological signals that cannot be monitored by traditional means, such as the impulses of the retinal nerves are becoming available [12]. Although flexible electronics exhibit extraordinary characteristics in collecting biophysical signals, they are still greatly limited in an attachment to joints and organs with large deformations. Direct use of wave/wrinkle [13,14,15], island-bridge [16,17,18], textile [19,20], or interlock [21,22] structures increases the stretchability of the electronics, but is still yet to maximize the strain without performance changes. Thus, there is an urgent need to develop a large-scale strategy of ultra-thin stretchable electronics.

The art of paper-cutting originated in the East, over time, it has become one of the brilliant and beautiful oriental arts throughout history. In recent years, it has brought new inspirations in art design, toys, architectures, engineering design [23,24], etc. Among them, in electronic engineering, the ancient art sliced through a roadblock with the development of flexible and elastic electronics. Structures such as kirigami and origami are applied in traditional planar electronic devices to realize the extension from two-dimensional to three-dimensional [25,26,27,28]. Such architecture can achieve several orders of magnitude of tensile strain, which is one of the most effective combinations of materials and structures characteristics [29,30,31]. Chinese paper-cutting art has a long history and is still being used today in a variety of festivals and ceremonies. In this report, we apply the traditional paper-cut structure to flexible electronic devices, realizing the excessive deformation of electronic devices. We verify the concept from many aspects such as experiment and mechanics and prove the ancient Chinese culture effectiveness and extraordinary guidance in the latest electronics.

Two-dimensional materials are being widely studied due to their excellent electrical properties, mechanical properties, and unique physical properties [32,33,34,35,36,37]. However, the zero-band gap of graphene excites the transition metal dichalcogenide (TMDC) emerging as a new research hotspot for two-dimensional semiconductor materials. In particular, TMDC forms a set of atomically thin layers of two-dimensional composites with superb mechanical property, which is ideal for essential flexibility [38,39,40]. Together with its high electrical and optical properties, high biocompatibility, and large surface to volume ratio [33,34,35,36], it is an excellent candidate for flexible electronics. However, in order to obtain a large piece of TMDC material with excellent performance is a very challenging task. The material prepared by the CVD method has poor electrical properties, while the traditional exfoliation method can only produce small size materials [41,42,43,44,45]. We first report a large flexible MoS_2_ device fabricated and transferred using a gold-assisted method; Here, we creatively use the gold-assisted top-down method to produce large-scale MoS_2_ nanosheet based on the large van der Waals force between gold and MoS_2_ [46]. A large-area, complete device-level paper-cut structure based on MoS_2_ is first realized. Although kirigami structures have been reported on electronic devices, [47,48,49] they are limited to single materials or devices with relatively small patch size in simple geometries. We first propose a super-deformable stretchable flexible device based on MoS_2_. Inspired by the traditional Chinese paper-cut structure, the large-size and large-deformation multi-functional electronic device is successfully developed, and the reliability of the device was proved through both experiments and mechanical calculation. It provides a new dimension for the TMDC-based large deformation bioelectronic device.

In our work, we designed a paper-cut patch with mechanically optimized hybrid architectures, which are consisted of a layer of electronic devices sandwiched by two layers of elastic polyimide. The active multifunctional sensor layer is based on MoS_2_ nanosheet and photo-patterned metal electrode, while the elastic polyimide is cut into kirigami structure for extreme stretchability and elasticity. Such an architecture places the functional components on both vertical and horizontal distribution and effectively protects electronics three-dimensional mechanical deformations. The hybrid sensor system collects optical and thermal information from the skin or the other organs. The MoS_2_-based device exhibits high performance in detecting optical and thermal signals. The design is further examined and optimized by modulating the shape and cutting size to ensure the performance of the device. 

## 2. Experiment and Method

Fabrication of devices on a rigid substrate: MoS_2_ is first exfoliated and transferred onto the SiO_2_/Si substrate. The active channel is then patterned with photolithography and SF_6_/O_2_ plasma etching (Oxford RIE), followed by evaporating a layer of Ti/Au. The metal layer is finally patterned and lift off to for the source and drain electrodes.

Fabrication of paper-cut electronics: The process starts with spin-coating (3000 rpm) a layer of poly(amic) acid (PI-2545, HD MicroSystems, Parlin, NJ, USA) onto a clean glass substrate. The PI layer is then cured on a hot plate at 150 °C for 10 min and 250 °C for 1 h to form the flexible substrate and isolation layer. After transferring the MoS_2_ nanosheet onto the bottom PI substrate with PDMS stamp, a layer of Au (150 nm) is evaporated by electron beam evaporation. Photolithography and wet etching are used to define the metal mesh as the electrode. MoS_2_ channel is protected and patterned with a thin layer of Cr (20 nm). A top layer of PI is covered on top of the active layer for isolation and protection. The two layers of PI are finally pattered with photolithography and reactive ion etching (O_2_ plasma, RIE) in the kirigami paper-cut geometries that match those of the metal traces. The entire system is further released from the glass substrate and floated on the water with buffered oxide etchant (BOE, Sigma-Aldrich, St. Louis, MO, USA).

Material characterization: MoS_2_ nanosheet is characterized by optical microscopy (AmScope, Irvine, CA, USA), Raman spectroscopy (Laser Quantum DPSS, 532 nm, Fremont, CA, USA), and TEM (FEI Titan 80–300, Hillsboro, OR, USA).

Electrical and optical characterization: Electrical characterization is conducted by a parameter analyzer (Agilent B1500A, Santa Clara, CA, USA) under ambient conditions. The UV light (365 nm) is illuminated and tuned with the Panasonic NUJ6170/NU6420 light source.

Simulation Method: To get a better understanding of the response of different kirigami structures, we perform Finite Element (FE) analysis on both unit cell and the whole structure by using the commercial package ABAQUS6.14/Standard [50]. Since the local strain among the structure is quite small and the plasticity has little effect on the deformation, we then use a linear elastic material model (with E = 4.33 GPa and ν=0.4) to capture the behavior of PI material. Comparing with the kirigami structure the Au layer is extremely thin which has little effect on the deformation and can be neglected. In all the simulations we discretize the structures using four-node general-purpose shell elements with reduced integration and hourglass control (S4R element type). Then the non-linear static simulations (* STATIC module in ABAQUS) are conducted with volume-proportional damping (using the option STABILIZE in ABAQUS) added in the model to facilitate the convergence, and set the dissipated energy fraction equal to 2e^−4^ and the maximum ratio of stabilization to strain energy equal to 0.05.

Calibration of the temperature sensor: The calibration is carried out in a boiled DI water bath. A platinum (Pt) based resistance temperature detector (RTD, CENTER 376, Taipei, Taiwan) is used as the temperature standard.

## 3. Result and Discussion

### 3.1. Concept and Inspiration of Paper-Cut Electronics

**Figure 1:**
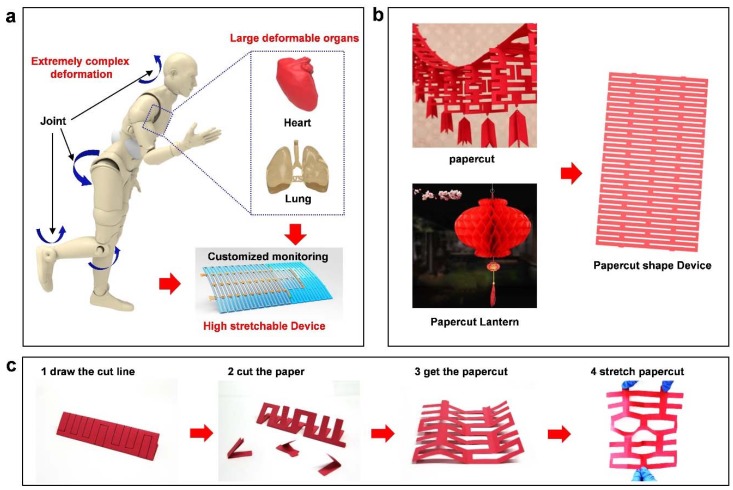
The design inspiration and concept of paper-cut electronics: (**a**) The application scenarios of kirigami structure devices, including highly deformed organs and complex deformed joints. (**b**) The design of paper-cut structure, inspired by traditional Chinese paper-cut art and paper-cut lanterns. (**c**) The process of cutting the paper-cut structure.

In order to continuously monitor physiology signal from joints with large movement and organs with complex deformation, a highly stretchable paper-cut device is fabricated (Figure 1a). Inspired by an ancient Chinese design of the structure paper-cut and a foldable paper-cut lantern, the structure can realize expansion from two dimensions to three dimensions (Figure 1b). The paper-cut art process is shown in Figure 1c, by cutting slit patterns and performs stretchability by the out-of-plane bending around each slit. Starting with patterning the paper by folding and drawing the cut line, the paper is then cut along the cutting line. Finally, the paper is unfolded to get the stretchable paper-cut structure. The stretching ability of the remaining parts dominates the flexibility of the entire system. The out-of-plane stretching properties introduce a much larger strain compared to the in-plane bending. 

### 3.2. Structure of the Paper-Cut Electronics

**Figure 2:**
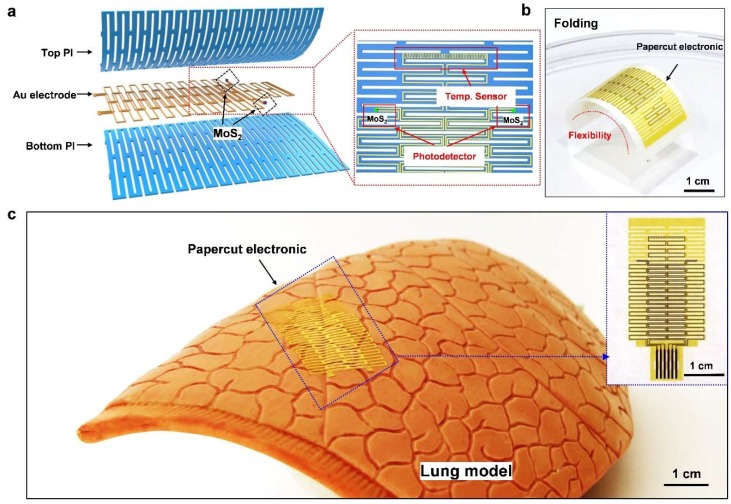
Structure of the paper-cut electronic device: (**a**) The structure architecture and distribution of the paper-cut electronic device, which consists of a sandwich structure comprising a temperature sensor and two symmetrical photoelectric sensors. (**b**) The device is attached to a piece of tape, and the photograph showing its bending characteristics. (**c**) Attachment of the device to the surface of the artificial lung.

Based on the paper-cut concept, a multifunctional electronic with the highly stretchable and deformable film is fabricated and presented. The paper-cut film is realized by patterning and etching optimized slits using conventional fabrication processes, containing a multifunctional hybrid sensor system. The entire electronics system is cut into paper-cut shape with the structure and the layout shown in Figure 2a. The system contains groups of single kirigami cells, while devices are placed on the center of the wing of the single cell to minimize the strain. The polyline shape gold mesh (150 nm) and the sensors are sandwiched by two layers of slit-designed kirigami polyimide (PI, 6 μm) film so that the effective layer lays in the middle of the system. Placing the high modulus layer on the mechanically neutral plane of zero stains can provide optimal stretchability [7,51,52,53]. Such PI-Au-PI hybrid structure is designed for mechanical support and electrical insulation. The system consists of three pairs of thin polyline shape metal interconnects for one temperature sensor and two symmetrical photodetectors. In order to maximize the functionality of the sensors under strain, the large-area thin and dangling-free active material MoS_2_ serves as the active material by gold mediated exfoliation method. Figure 2b shows the extreme bending property of the entire system by laying on a piece of tape, while the device also shows extreme lightweight after released and floating on the surface of the water (Figure S1). The photo of the hybrid paper-cut electronic placed on an artificial lung is presented in Figure 2c with perfect adhesion and attachment to the ravine on the organ surface. 

### 3.3. Exfoliation Method and Electrical Characterization of the Sensing Layer

**Figure 3:**
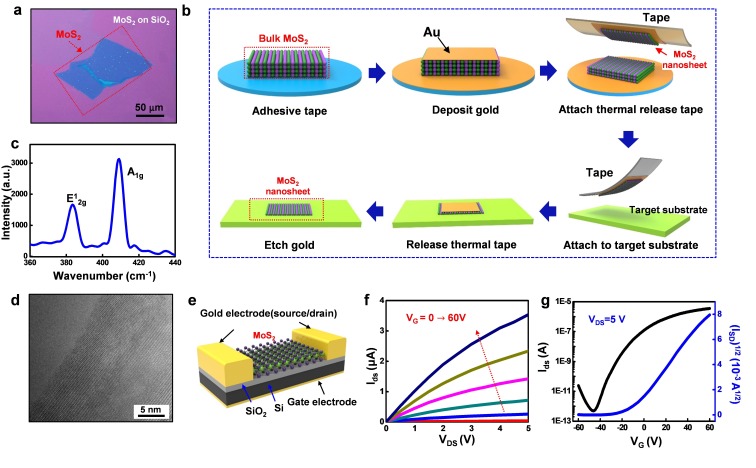
Transfer process, material and electrical characterization of the MoS_2_ nanosheet: (**a**) Optical image of exfoliated few-layer MoS_2_ with the lateral size of ~120 nm. (**b**) Illustration of the gold-mediated exfoliation process. (**c**) Room-temperature Raman spectrum of few-layer MoS_2_. (**d**) High-resolution TEM images of the atomic layer of MoS_2_. (**e**) Three-dimensional schematic view of the back-gated transistor on the SiO_2_/Si substrate. (**f**) A typical output curve (I_DS_ vs. V_DS_) with the back-gate voltage V_G_ sweep from 0 to 60 V. (**g**) Transfer curve (I_DS_ vs. V_G_) with V_DS_ = 5 V, the black curve shows the log scale, the blue curve is the square root of I_DS_.

TMDC, one of the most studied two-dimensional semiconductor materials filling up the family of the flexible sensing materials because of its superior mechanical properties [54,55]. Previous methods, including top-down and bottom-up methods, were either small in size (area size smaller than 100 μm^2^) or bad in performance, which was less ideal for conducting flexible devices. We report an advanced exfoliation method to obtain large MoS_2_ nanosheet with high performance, which optical image is shown in Figure 3a. A single large uniform nanosheet (lateral size ~120 um) is acquired by this method. The process is illustrated in Figure 3b [46]. The gold-assisted exfoliation method shown in the figure starts from evaporating over 100 nm of gold on top of the MoS_2_ bulk. The strong van de Waals force between gold and S atom assists detaching the topmost layer from the bulk by a thermal release tape. The tape with a layer of gold and the MoS_2_ top layer is then attached to the target substrate. After the tape is released with heat and the substrate is cleaned by the gold etcher (KI/I_2_), the large and thin MoS_2_ nanosheet is finally transferred onto the target substrate for further fabrication or pick up. The MoS_2_ nanosheet on Si/SiO_2_ substrate is first characterized by Raman spectroscopy (Figure 3c) and transmission electron microscopy (TEM) (Figure 3d). The A_1g_ mode at 409 cm^−1^ and the E^1^_2g_ mode at 382 cm^−1^ from Raman is consistent with the typical vibration modes of few-layer MoS_2_ [56].

The electrical properties of the material are characterized by a back-gated field effect transistor (FET), with its fabrication process illustrated in Figure S2 and described in the “Experiment and Method” section. The metal contacts are patterned on the top of the MoS_2_ channel working as the source and drain electrodes. A layer thickness of 285 nm SiO_2_ is on the substrate, serving as a gate oxide. The FET has finally connected with p doped Si as a back gate. According to the described design, the 3D schematic of an FET is shown in Figure 3e. By collecting the electrical properties of the MoS_2_ nanosheet, the ability of it applied in electronics is evaluated. An output characteristics curve (I_DS_ vs. V_DS_ with varied V_G_) (Figure 3f) is performed to describe the gate dependency of the device. In the test, the gate voltage is swept from 0 to 60V with a step of 10V. The device approaches the saturation region when V_DS_ = 4 V. The typical transfer curve (I_DS_ vs. V_G_, black for log scale, blue for I_DS_^1/2^) at V_DS_ = 5 V (Figure 3g) are observed to obtain the ON/OFF ratio (>10^7^) and the variation of the threshold voltage. From the transfer curve and the square root of I_DS_, the field effect mobility and the threshold voltage can be calculated with $I_{\mathrm{DS}}=\frac{\mathrm{WC}}{2L}\mu_{\mathrm{sat}}{\left(V_{G}-V_{\mathrm{th}}\right)}^{2}$ [57] where W and L are the channel width (5 μm) and the length (5 μm), μ_SAT_ is the saturation mobility, C is the capacitance of the gate dielectric, and V_th_ is the threshold voltage. The device exhibits a high ON-currents (10 µA µm^−1^ at V_DS_ = 5 V), a high field-effect mobility of 11.95 cm^2^/Vs and a threshold voltage of −20 V. The typical mobility for MoS_2_ is thickness dependent in the range of 0.06 cm^2^/Vs to 70 cm^2^/Vs [58]. The device created with gold-assisted exfoliated MoS_2_ shows solid electrical performance for electronic applications.

### 3.4. Fabrication Process and Device Characteristics

**Figure 4:**
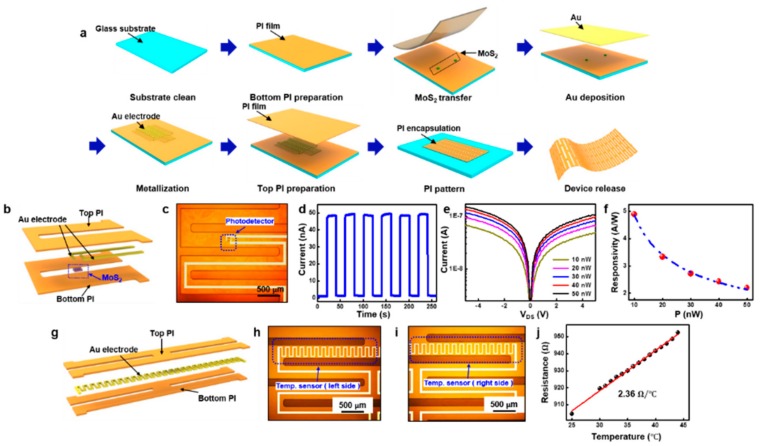
‘Pattern-cut-release’ fabrication process and the device characterization: (**a**) Schematic illustration of the fabrication process. (**b**) Three-dimensional schematic view and (**c**) optical image of the MoS_2_ based photodetector. (**d**). Time-dependent pulsed photoresponse of the device under UV light (λ = 365 nm), V_DS_ = 5 V and P = 10 nW. (**e**) Source-drain characteristic of the photodetector under different light intensity (10 nW ~ 50 nW). (**f**) Photoresponsivity versus incident power. (**g**) Three-dimensional schematic view of the temperature sensor. (**h**) Left side and (**i**) right side optical image of the temperature sensor. (**j**) Temperature-dependent resistance curve for the thermometer.

To generate MoS_2_-based paper-cut electronic devices with extreme flexibility, elasticity, and softness, we introduce a pattern-cut-release fabrication process whereby first the polyimide (PI) and the active layer are patterned, followed by PI cutting using a conventional plasma etching and releasing with buffered oxide etching (BOE) (Figure 4a). A clean glass substrate is first prepared as a temporary carrier, coated by an insulation layer of polyimide (PI) (~2.5 μm) with spin-coating and curing (150 °C for 10 min, then 250 °C for 1 h). In order to place the MoS_2_ nanosheet accordingly, the flake is transferred onto the PI layer by a traditional transfer printing process with a poly(dimethylsiloxane) (PDMS) stamp [59]. A thin Cr mask is used for protection so that the SF_6_/O_2_ plasma can be applied to etch the material. The gold interconnect layer is evaporated on top of the PI film, followed by patterned into the polyline shaped electrodes. Another layer of PI as encapsulation and insulation is covered on top of the active layer, followed by patterned with oxygen plasma into paper-cut hybrid structure to match the metal traces. Such procedure forms a sandwich structure so that the active layer is placed in the neutral-mechanical plane. The entire device is finally cleaned and chemically released with buffered oxide etchant (BOE) from the glass carrier.

The multifunctional device composed of two photodetectors and a temperature sensor with a detailed geometric design is illustrated in Figure S3. The electronic devices are tested with anisotropic conductive film (ACF) cables (100 μm, Elform), which is interconnected with a print circuit board (PCB) with the 500um gap between pins (Figure S4). The 3D schematic structure and the correspondence optical image of the MoS_2_-based photodetector are shown in Figure 4b,c. Based on the characterization test under UV (λ = 365 nm) illumination, the optical properties of the photodetector is examined [60]. We first recorded the pulsed photoswitching time-dependent curve at V_DS_ = 5 V (Figure 4d). The excited electrons from the illumination of light generate the photocurrent under source-drain bias, resulting in an increase of the source-drain current. The fast response (300 ms) and recovery time (300 ms) are reported with a high ON/OFF ratio of over 100. The photodetector is then characterized under the illumination of different intensities from 10 nW to 50 nW to obtain the photoresponsivity level. (Figure 4e) Trends on the curves highlight the effect of incident illuminance to the photocurrent, with the light current increases from 48.9 nA to 109.7 nA while the intensity increases from 10 nW to 50 nW. The photoresponsivity is defined as the current–power relevance as the ratio of the photocurrent variance and the incident power:$\;R=\frac{I_{light}-I_{dark}}{P_{0}}$ [61], Where $P_{0}$ is the incident power, $I_{light}$ and $I_{dark}$ are the light and the dark current. The photoresponsivity regarding the input power is extracted in the Figure 4f. As shown in the figure, the photoresponsivity decreased from 4.9 A/W to 2.2 A/W. There is a saturation region with high incident power due to the trap states and the reduced available states of the large surface of the TMDC [34]. The band diagram of the photodetector is shown in Figure S5 to illustrate the photon generation under illumination. The device starts from its equilibrium state and forms a small Schottky barrier height at the metal contacts in the dark state. By applying a source-drain voltage, the electron–hole pair generated by the light absorption can be split with the flow of the electrons. In the design, two photodetectors are place symmetrically to map deferent positions and increase the robustness of the system. 

Body temperature is one of the most crucial parameters, which can reflect a lot of physiological index of the human body. For example, the variation of the body temperature daily can indicate fever, septicemia, inflammation, or ovulation. In the meantime, the environmental temperature is also playing a significant role in daily life. Thus, the continuous monitoring of the body temperature and the environmental temperature is extremely important. As a result, a large demand for accurate, highly efficient, and lightweight thermistors on an e-skin patch is requested in biomedical applications.

The three-dimensional schematic of the temperature sensor is shown in Figure 4g, which consists of a gold wire in between two layers of the PI. The left and right side of the thermometer is captured with an optical microscope (Figure 4h,i). Gold has a linear dependence to temperature in a certain range, where $R=R_{ref}\left(1+\alpha \left(T-T_{ref}\right)\right)$. α is the temperature coefficient, $R_{ref}$ is the reference resistance, $T-T_{ref}$ is the variation of the temperature, and *R* is the temperature dependent resistance. Based on this mechanism, a conductor can be applied to precisely sense subtle temperature change by the electrical signal. The device is measured from room temperature to 45 °C in order to be precise in body temperature. The temperature versus resistance curve of the device is presented in Figure 4j, which is good in linearity and has a sensitivity of 2.36 Ω/°C. The result indicates that out sensor can measure the body temperature in a full range with precise and linear response, which meets the satisfaction of the thermometer in daily life. 

### 3.5. Optimization of the Kirigami Structures

The described fabrication process enables the functionalization of the kirigami structure device to be free of shape and cut design. As is well-known, cut slits within different direction and shape on the kirigami structure can produce hundreds of 3D geometries. Here, we studied three different kirigami structures (linear, triangular, and trapezoidal) to compare the stretchability of the film and optimize the strain, which the geometry distribution is illustrated in Figure S6. Linear cut means that each slit is horizontal to the direction of the stretch, which generates an out-of-plane force along the *x*-axis under the strain. The second structure, triangular shape patterns the film with diagonal slits and form a pyramid shape under strain. We also compared a trapezoidal geometry with a combination of linear and triangular shape. This structure twists the horizontal line to the opposite direction to form a 3D architecture. 

**Figure 5:**
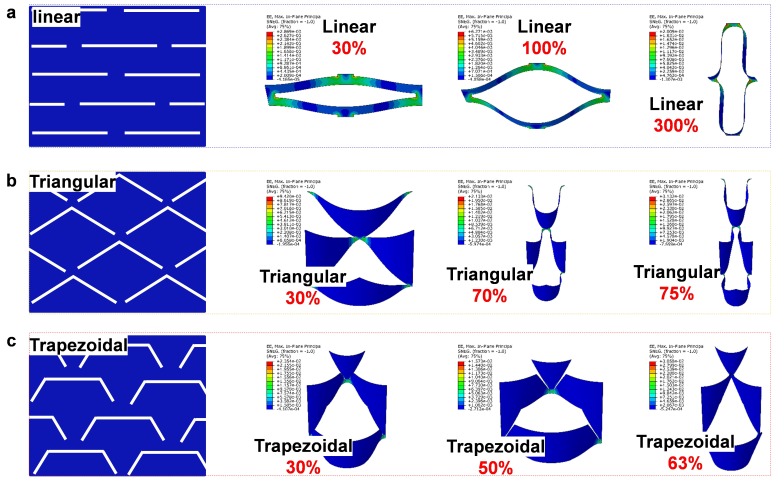
Design optimization of the structure, with white represents the cutting slit and blue represents the substrate: (**a**) Linear structure with tensile strain of 30%, 100%, and 300%. (**b**) Triangular shape with tensile strain of 30%, 70%, and 75%. (**c**) Trapezoidal structure with tensile strain of 30%, 50%, and 63%.

Kirigami inspired metamaterials are widely investigated due to their ability to achieve high levels of deformation without destroying the base materials. The kirigami structures can be very different for different applications [62]. In our case, we need the global strain of the device as large as possible and the maximum local strain should be less than 3% to avoid the noise generated by the deformation during the tests. To figure out a rational design among all different kirigami cuts/patterns, we select three widely used kirigami patterns (linear, triangular, and trapezoidal cuts, geometries can be found in SI) and, then conduct Finite Element (FE) simulations to compare the local strain with different applied strain in Figure 5a–c. Guiding by the simulation results, we found that the linear cut structure can support much more deformation than others. Specifically, when the maximum local strain is less than 3%, the linear cut kirigami structure can achieve more than 300% global strain while the triangular and trapezoidal cut structures can only support up to 75% and 63%, respectively. So according to the FE results, we choose the linear cut as the base structure of our device and smooth the cuts by fillets to reduce the stress concentration around the ligaments.

### 3.6. Stretchability and Flexibility Test

**Figure 6:**
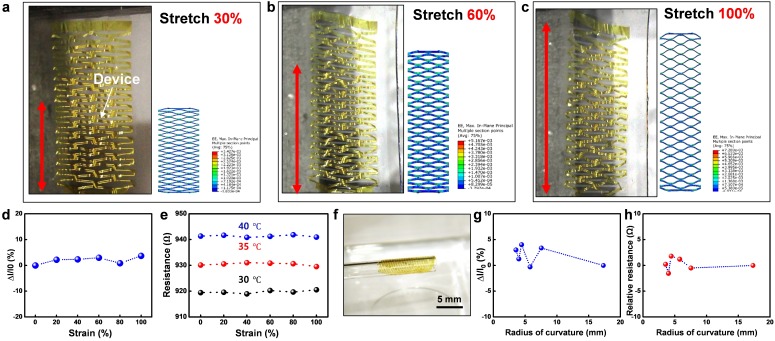
Stretchability and flexibility of the device: (**a**) Optical image and FEA of the device under 30% tensile strain. (**b**) Optical image and FEA of the device under 60% tensile strain. (**c**) Optical image and FEA of the device under 100% tensile strain. (**d**) Photocurrent change regarding the tensile strain up to 100%. (**e**) Resistance versus strain under temperatures of 30 °C, 35 °C, 40 °C. (**f**) The device is wrapped around a glass tube. (**g**) Photocurrent versus bending radius from 3.5 mm to 17.3 mm. (**h**) Resistance change of the temperature sensor under bending radius from 3.5 mm to 17.3 mm.

In this section, we further test the stretchability and the flexibility of the device under different tensile strain and bending radius. To demonstrate the stretchability of the device, we stretch a fabricated device with a strain of 300% by tweezers on the water (Figure S7) and test it on a uniaxial tension test machine with the applied strain from up to 100%. As predicted by the FE simulations, the device exhibits very high levels of deformation without introducing any mechanical damage in the structure (See Figure 6a–c). The electrical signal for the devices under different strain is also performed to further understand the effect of the stretch to the electronic device. The performance of the photodetector and the temperature sensor are both tested under strain by their current change and resistance change, respectively. The relative change of the photocurrent is less than 3% under strain, while the temperature sensor remains stable under different strain and temperature. Concluded from Figure 6d,e, the devices exhibit high stretchability and abide by their readings under the tensile strain from 0 to 100%. 

Besides the stretchability, the flexibility and softness are also the most substantial factors for precision healthcare especially on the organs and tissue with small modulus and high deformation. In order to show the flexibility of the device, the patch is first picked up with a glass test tube (Figure 6f). The system is fully twisted all over the tube after picked up from the water and is floated when placed back. With the same method, the device is verified by its electrical signal with different bending radii by various tubes (Figure S8). Figure 6g,h represent the relative current and resistance change for the photodetector and the temperature sensor. The current varies less than 4% for the photodetector while the resistance changes less than ±2 Ω under a tensile strain up to 100%. With a smaller bending radius, the out-of-plane bending dominates the electrical properties.

### 3.7. Applications

**Figure 7:**
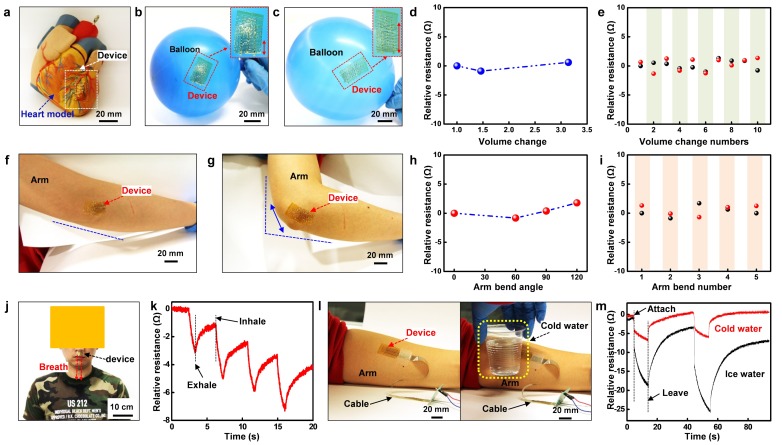
Applications and test: (**a**) Device attached to an artificial heart. (**b**) Device pasted on a less inflated balloon. (**c**) The inflation of the balloon, with the stretch of the device. (**d**) Resistance change of the temperature sensor with the balloon expands its volume. (**e**) Relative resistance under expansion cycles up to 10 (black dot: before expansion, red dot: after expansion). (**f**) The original state of the device pasted on the elbow. (**g**) Arm bent to 90°, with the device stretched along with the bending. (**h**) Relative resistance under different bending angles. (**i**) Relative resistance when bending up to 5 cycles (black dot: before bending, red dot: after bending). (**j**) The photograph of the device under the nose to monitor the breathing. (**k**) Time-dependent relative resistance in regarding the inhale and exhale cycles. (**l**) The device on the arm is tested with cold water. (**m**) Time-dependent response of the signal in touch with cold water (red) and ice water (black).

As described before, highly stretchable, flexible, and soft devices are ideal for organs and tissues with large inflation and joints with high deformation. The first challenge is collecting physiology signals from the heart, which is super soft, has an unregular shape, and changes rapidly in volume as it pulses. Recent research has demonstrated flexible, wearable, and disposable cardiac biosensors [6] and symbiotic cardiac pacemaker [63] placed on the heart. However, a more rigorous requirement of such a device is that the film must be ultra-soft to match the extreme low modulus of the delicate organs. Other requirements include seamless attaching and high stretchability under strain. We first demonstrate the device attached to an artificial heart, where the metal trace follows the artificial blood vessel tightly (Figure 7a). To verify stretchability with the expansion of the heart, the device is tested with a balloon (Figure 7b,c). When pasted on the balloon, the structure fits itself to the surface with the inflation. The temperature sensor is further examined under different expansion ratios and times. Figure 7d shows the variation of the resistance under the volume expansion ratio of 1.46 and 3.14, suggesting that the resistance change less than 2%. Because of the continuous pulse of the heart, the device is also tested with expansion up to 10 times. Figure 7e describes the phenomenon that each beat causes a neglectable change (<3%) on the resistance.

Another challenge for such design is the application on joints with high deformation including twisting and bending. Due to the deformability of the joint, the device should be able to bend over 90° consequently. Thus, the patch is pasted on the elbow to measure its bendability (Figure 7f,g). Figure 7h,i are presenting the electrical properties of the resistance with a bend angle up to 120° and 5 repeat bends. The result concludes that with a large bending angle and multiple bending times, the resistance changes less than 3%, which is reliable for joint placement. 

With such advantages, the device patch can be applied to health care and electronic skin, by continuous monitoring breathing period or sensing the environmental temperature exchange. Sleep disorder or obstructive sleep apnea-hypopnea syndrome (OSAHS) are both important medical conditions that affect sleep. They can be severe for patients. Continuous monitoring breath rhythm helps to understand and diagnosing such condition and preventing related disease. The device is thus pasted under the nose to acquire signals under breathing (Figure 7j). The four cycles of pulsed resistance change are shown in Figure 7k, with resistance with each exhalation. Inhalation, on the contrary, brings the resistance back. By recording the period and the level of breathing during sleep, a more precise analysis of health condition can be done throughout a day. Based on the super sensitivity of the device, it can also be applied to sense the change of the environment temperature as an e-skin. By attaching to the skin, the device reads the skin temperature. Touched with lower temperature objectives will bring significant change to the signal (Figure 7l). Figure 7m quantities the resistance change under ice water and room temperature water, where it indicates the different temperature exchange sensed by e-skin. 

## 4. Conclusions

In conclusion, flexible and stretchable electronics provide a good opportunity to perform biomedical research on joints and other highly deformed organs. Here we proposed a non-invasive, versatile, high-performance, inexpensive, intelligent, and stretchable electronic system based on innovative hybrid structures and two-dimensional nanomaterials. MoS_2_ is characterized by its material and electrical properties to confirm the performance. The traditional Chinese paper-cut structure guarantees the high tensile strain and comfortability of the device, where the flexibility, bendability, and stretchability are demonstrated and optimized. With a paper-cut based film design, we fabricated multifunctional and flexible electronic devices, and demonstrated optical and thermal signal recordings from the joint and the skin. We also proposed a few applications in healthcare and environmental monitoring. Employing high stretchability and flexibility, devices on highly deformed organ and the movable joint can be displayed in a new class of applications in the precision healthcare electronics.

## Supplementary Materials

The following are available online at https://www.mdpi.com/2079-4991/9/7/922/s1, Figure S1. The paper-cut flexible electronic floating on the water, indicating its superior lightweight and softness; Figure S2. The fabrication process of the MoS_2_ based back gate transistor on the rigid substrate: (a) prepare a clean SiO_2_/Si substrate. (b) Exfoliate and transfer the MoS_2_ nanosheet on the substrate. (c) Pattern the MoS_2_ active channel. (d) Deposit a Ti/Au layer, followed by patterning into electrodes; Figure S3. Detailed layout of the design: (a) The CAD structure of the system (Blue trace is the metal mesh, red trace is the PI). (b) Detailed design of the temperature sensor, with a cross-section view in the corner. (c) Detailed design of the photodetector, with a cross-section view in the corner; Figure S4. The paper-cut flexible electronic is connected with an external PCB(FPC-24P) with a heat seal ACF cable; Figure S5. The mechanism of the MoS_2_ based photodetector: (a) The band diagram of MoS_2_ and Au, in correspondence to vacuum level. (b) The band alignment between the two electrodes and the MoS_2_ layer. (c) Under a bias, the band bends according to the bias direction so that the electrons exited from the light illumination are moved to the conduction band; Figure S6. The detail geometries of the three different cutting patterns. From top to down: linear, triangle and trapezoidal; Figure S7. Stretching the device on the water with a tweezer: (a) The original state of the device. (b) The stretched device with tensile strain of 300%. (c) Photograph of the top part of the device; Figure S8. The bendability test: (a) Photograph of the device bending on the stick of a cotton swab. (b) Testing with different bending radius.
