# Supplementary material for: Paper-Cut Flexible Multifunctional Electronics Using MoS2 Nanosheet
Source: Nanomaterials (Basel). 2019 Jun 26;9(7):922. doi: 10.3390/nano9070922 (PMC6669538; doi:10.3390/nano9070922)
Supplement: Supplementary file 1 [file nanomaterials-09-00922-s001.pdf]

Supporting Information

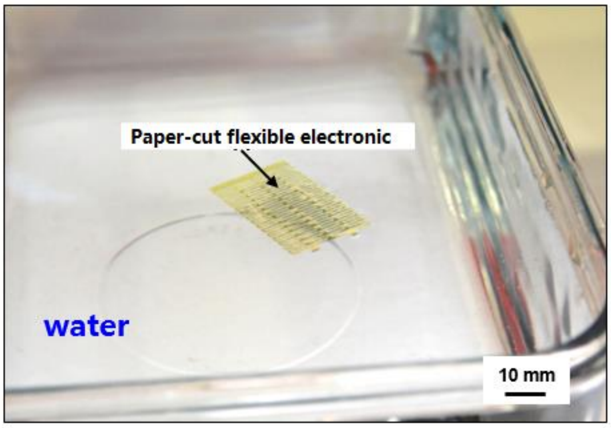

Figure S1. The paper-cut flexible electronic floating on the water, indicating its superior lightweight and softness.

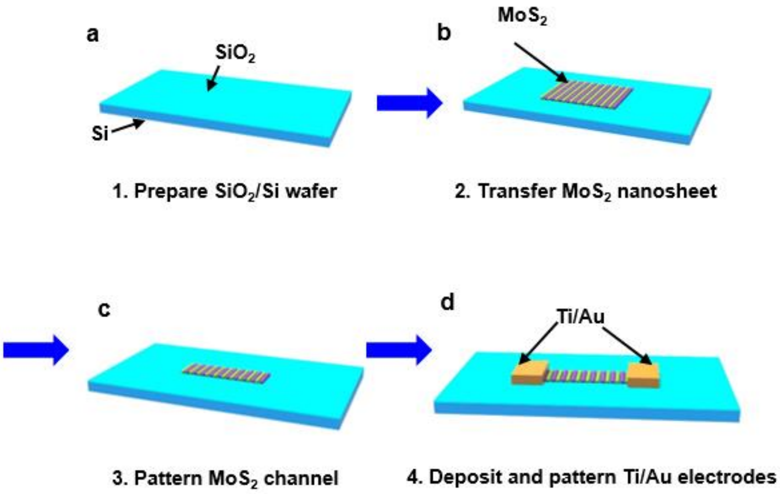

Figure S2. The fabrication process of the MoS_2 based back gate transistor on the rigid substrate: (**a**) prepare a clean SiO_2/Si substrate. (**b**) Exfoliate and transfer the MoS_2 nanosheet on the substrate. (**c**) Pattern the MoS_2 active channel. (**d**) Deposit a Ti/Au layer, followed by patterning into electrodes.

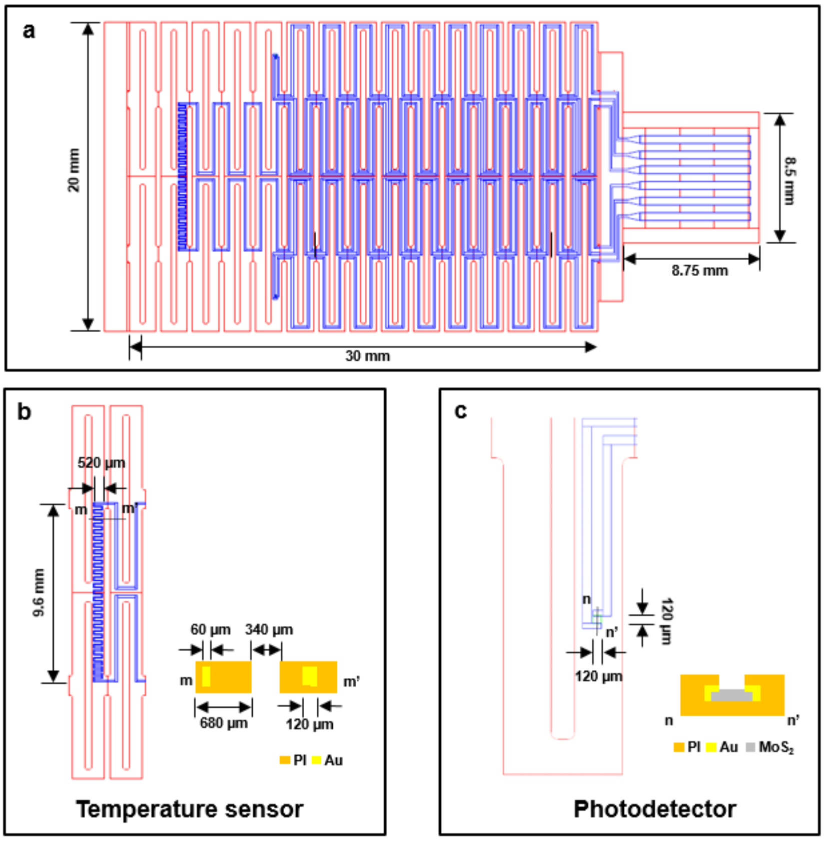

Figure S3. Detailed layout of the design: (**a**) The CAD structure of the system (Blue trace is the metal mesh, red trace is the PI). (**b**) Detailed design of the temperature sensor, with a cross-section view in the corner. (**c**) Detailed design of the photodetector, with a cross-section view in the corner.

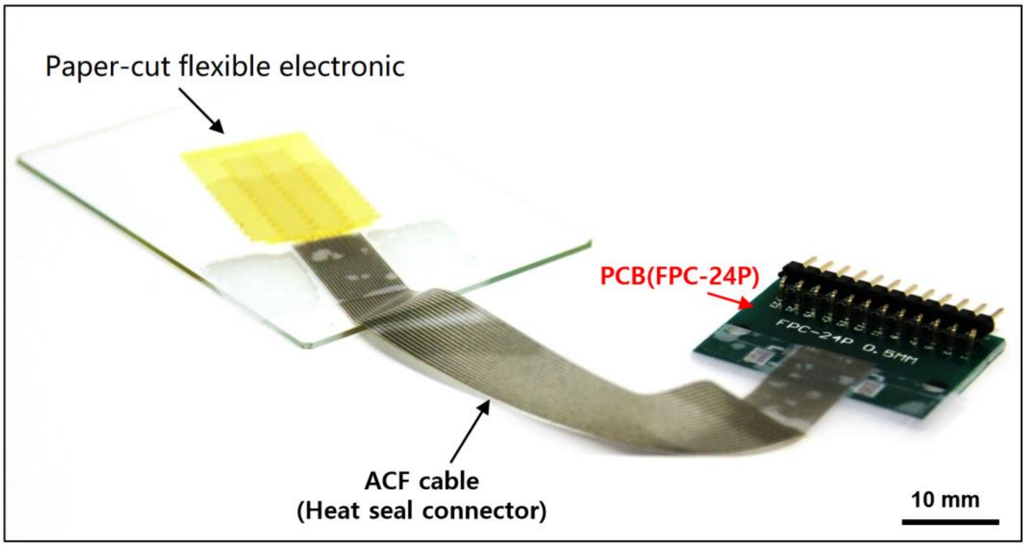

Figure S4. The paper-cut flexible electronic is connected with an external PCB(FPC-24P) with a heat seal ACF cable.

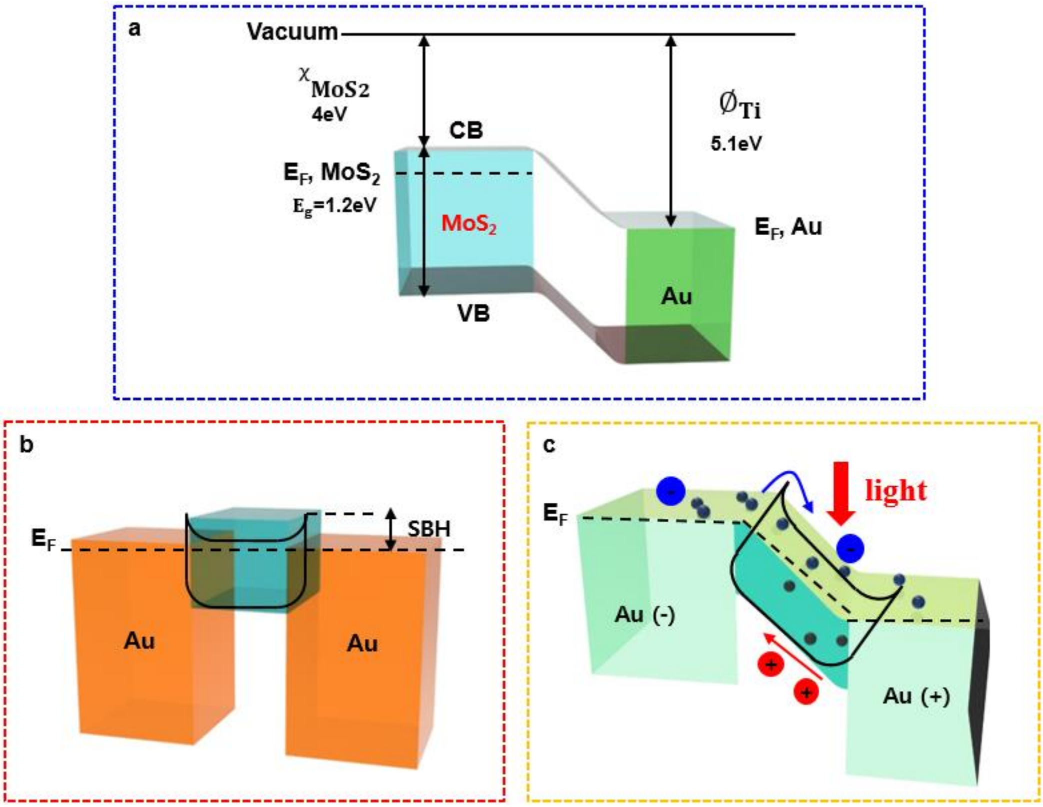

Figure S5. The mechanism of the MoS_2 based photodetector: (**a**) The band diagram of MoS_2 and Au, in correspondence to vacuum level. (**b**) The band alignment between the two electrodes and the MoS_2 layer. (**c**) Under a bias, the band bends according to the bias direction so that the electrons exited from the light illumination are moved to the conduction band.

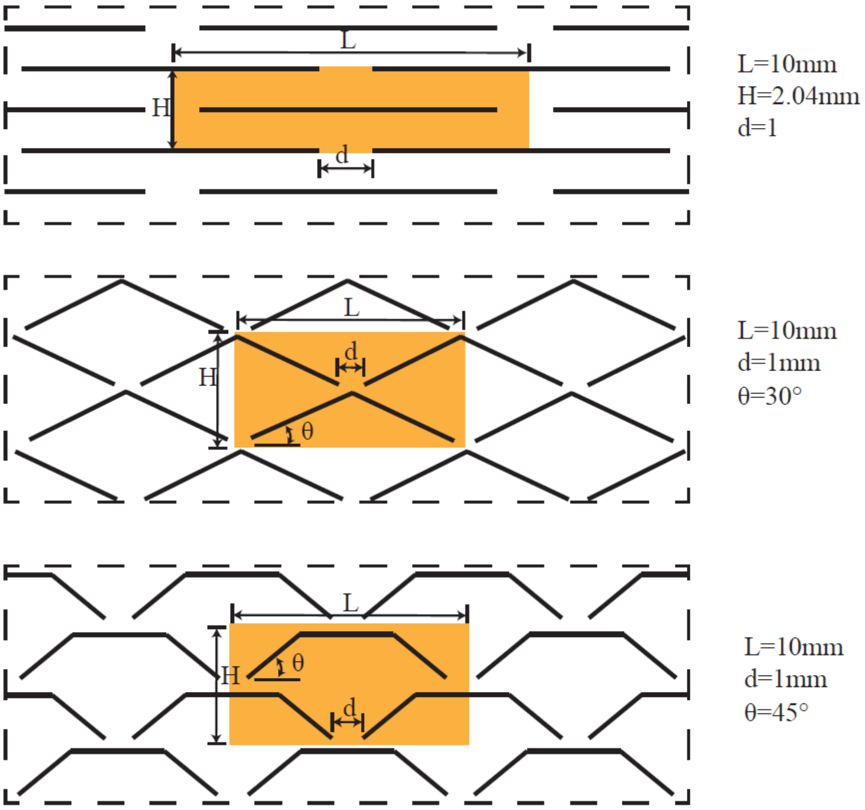

Figure S6. The detail geometries of the three different cutting patterns. From top to down: linear, triangle and trapezoidal.

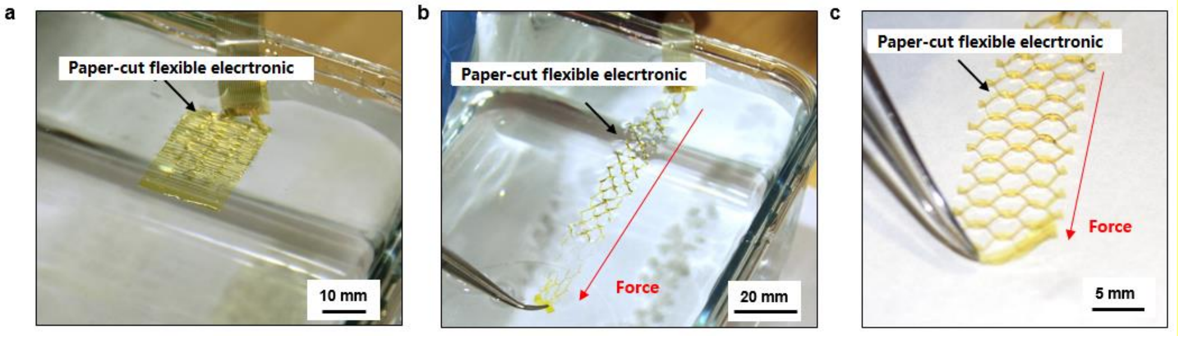

Figure S7. Stretching the device on the water with a tweezer: (**a**) The original state of the device. (**b**) The stretched device with tensile strain of 300%. (**c**) Photograph of the top part of the device.

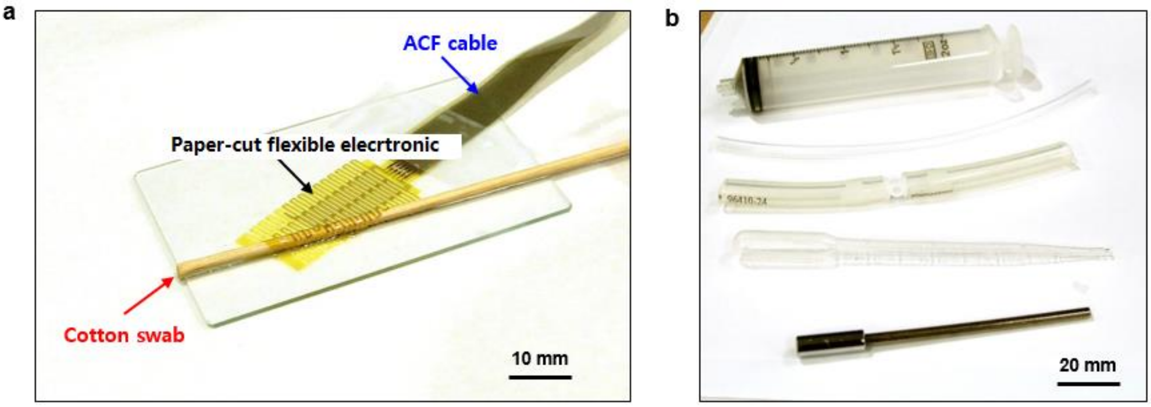

Figure S8. The bendability test: (**a**) Photograph of the device bending on the stick of a cotton swab. (**b**) Testing with different bending radius.
